# Alzheimer’s disease: the role of extrinsic factors in its development, an investigation of the environmental enigma

**DOI:** 10.3389/fneur.2023.1303111

**Published:** 2023-12-06

**Authors:** Swathi Suresh, Ankul Singh S, Rapuru Rushendran, Chitra Vellapandian, Bhupendra Prajapati

**Affiliations:** ^1^Department of Pharmacology, SRM College of Pharmacy, SRM Institute of Science and Technology, Kattankulathur, Tamil Nadu, India; ^2^Shree S. K. Patel College of Pharmaceutical Education and Research, Ganpat University, Kherva, Gujarat, India

**Keywords:** Alzheimer’s disease, environmental contaminants, air pollution, ozone, plastic, bisphenol A, pesticide, metal toxicity

## Abstract

In the realm of Alzheimer’s disease, the most prevalent form of dementia, the impact of environmental factors has ignited intense curiosity due to its substantial burden on global health. Recent investigations have unveiled these environmental factors as key contributors, shedding new light on their profound influence. Notably, emerging evidence highlights the detrimental role of various environmental contaminants in the incidence and progression of Alzheimer’s disease. These contaminants encompass a broad spectrum, including air pollutants laden with ozone, neurotoxic metals like lead, aluminum, manganese, and cadmium, pesticides with their insidious effects, and the ubiquitous presence of plastics and microplastics. By meticulously delving into the intricate web connecting environmental pollutants and this devastating neurological disorder, this comprehensive chapter takes a deep dive into their involvement as significant risk factors for Alzheimer’s disease. Furthermore, it explores the underlying molecular mechanisms through which these contaminants exert their influence, aiming to unravel the complex interactions that drive the pathogenesis of the disease. Additionally, this chapter proposes potential strategies to mitigate the detrimental effects of these environmental contaminants on brain health, with the ultimate goal of restoring and preserving typical cognitive function. Through this comprehensive exploration, we aim to enhance our understanding of the multifaceted relationship between neurotoxins and Alzheimer’s disease, providing a solid foundation for developing innovative *in-vivo* models and advancing our knowledge of the intricate pathological processes underlying this debilitating condition.

## 1 Introduction

### 1.1 Brief overview of Alzheimer’s disease

Dementia is a progressive cognitive decline that affects daily life but is not inevitable with age. The WHO estimates that 50 million people globally have dementia, with 10 million new cases annually. Dementia will affect 78 million by 2030 and 139 million by 2050. The major cause of dementia is Alzheimer’s disease (AD), with 50 to 75% of cases. It is the most common kind of dementia in older people, doubling every 5 years after 65 (1). AD is characterized by age-related cognitive and functional deterioration. This condition is associated to brain changes like the formation of fibrillary amyloid-beta (Aβ) neuritic and senile plaques. The production of β-pleated Aβ aggregates outside of neuronal cells is linked to tau protein hyperphosphorylation. Tau protein should stabilize neurons and allow nutrition transport. Hyperphosphorylation causes neurofibrillary tangles (NFTs) in neurons in AD. NFTs impede nutrition transport and cause neurotoxicity. This book chapter discusses environmental risk factors for AD pathogenesis and their molecular mechanisms. Understanding how environmental factors cause dementia is important for public health because it can inform community planning guidelines. Such knowledge may also aid national economies.

### 1.2 Importance of understanding AD development

AD is caused by a variety of genetic, environmental, and health variables, not just genetic problems. Age is the biggest risk factor for AD, but persistent environmental variables can significantly modify brain physiology, especially in LOAD, which usually emerges after 65. FAD commonly has a dominating Mendelian inheritance pattern. SAD, however, has no clear inheritance pattern. SAD may be caused by a mix of genetic and environmental factors rather than specific genetic abnormalities (2). Family genetics account for most EOAD instances. These cases are caused by APP, PSEN1, or PSEN2 mutations. LOAD is usually scattered, with no apparent familial inheritance pattern. Recently developed genome-wide association studies and sequencing (GWAS) have discovered over 20 SAD risk loci. However, the fact that many AD cases are not inherited shows the illness’s complexity (3).

### 1.3 Thesis statement: investigating the role of extrinsic factors in AD development is critical for a comprehensive understanding of the disease

The amyloid beta peptide has been implicated in its etiology, however the chemical mechanism is yet unknown. While folded as a monomer, A tends to self-assemble into amyloid fibrils. Mature fibrils can create oligomers, intermediate-sized aggregates that accelerate neuronal death, suggesting that this aggregation process is linked to the generation of strongly neurotoxic species. The biological significance of protein aggregation must be understood by understanding the formation of both the final fibrillar aggregates and these oligomeric stages. Protein aggregation resembles nucleated development in many respects. Soluble peptides undergo primary nucleation, which produces the first aggregates, then monomers are added in an elongation step to expand them. It has become clear that primary nucleation is often slow and that most new aggregates are formed in autocatalytic secondary processes involving existing fibrils, such as fragmentation or monomer-fibril interactions. Twofold nucleation using primary nucleotides and secondary processes best describes the aggregation data for the two major A variants. Highly poisonous secondary nucleotides have been identified (4).

A review of risk factors offers insights that can steer future research directions by compiling and evaluating the body of existing knowledge. Healthcare practitioners will find this information useful as well, as it can support early diagnosis, focused treatments, and individualized treatment regimens. Understanding the risk factors linked to Alzheimer’s disease also has important ramifications for public health. It makes it possible to create educational programs and public health strategies that target population-level risk factor reduction, awareness-raising, and general community improvements to brain health.

## 2 Data source and search strategy

A comprehensive review was conducted to explore peer-reviewed studies investigating the impact of risk factors (both environmental and non-environmental) on AD. Multiple electronic databases such as Science Direct, ProQuest, Google Scholar, DOAJ, PubMed, Scopus, and DOAJ, were searched using consistent search criteria. The search was restricted to English language publications without imposing temporal limitations. Specific search terms related to AD and risk factors were used across all databases, with slight variations in the search strategy tailored to each database. The search, conducted on August 5th, 2023, focused on the keywords ‘Alzheimer’s AND Risk factors.’ From the retrieved records, only those emphasizing the significance of any kind of risk factors, be they environmental or non-environmental, were included for analysis.

## 3 Genetics and Alzheimer’s disease

### 3.1 Overview of genetic risk factors

A human’s biological blueprint is encoded in 30,000 genes on 23 chromosomes. This interactive diagram shows the three genes responsible for familial AD and the chromosome with the most risky gene. Researchers discovered the first gene mutation that caused familial AD in 1987: amyloid precursor protein (APP).

### 3.2 Familial AD and early-onset AD

The main types of AD are familial and sporadic. FAD is marked by significant parietal atrophy, white matter abnormalities, and high Aβ production. It often causes early-onset AD. Only about 5% of AD cases are familial, caused by autosomal mutations in APP, PSEN1, or PSEN2 genes in the α-secretase complex. SAD, linked to late-onset AD (LOAD), is characterized by decreasing hippocampus volume and increased disease prevalence, including diabetes, obesity, and impaired Aβ clearance (5). Only a few hundred families worldwide contain the rare Alzheimer’s genes. These genes cause familial early-onset AD, which usually develops between 40 and 50. Most Alzheimer’s patients are above 65. The genes that cause “familial Alzheimer’s” are rare, but their discovery has helped us understand this illness. All of these genes produce or process plaque-forming beta-amyloid. Beta-amyloid may cause brain cell death and degeneration. Aducanumab (Aduhelm®) and Lecanemab (Leqembi®) may delay cognitive and functional decline in early Alzheimer’s patients by removing brain amyloid. Discuss healthcare expenses and availability with your provider. Additional amyloid-targeted drugs.

### 3.3 Common genetic variants associated with late-onset AD (e.g., APOE)

Over the past decade, scientists have found hundreds of Alzheimer’s genes. Scientists have identified over 70 AD-related DNA regions. Identifying the genes and their functions may help develop new dementia prevention, delay, and treatment methods. The APOE gene is a well-known gene that predicts AD. The APOE gene produces a protein that transports cholesterol and other lipids in the circulation. This procedure’s shortcomings may cause AD. There are several APOE alleles (2, 3). Some evidence suggests APOE 2 confers disease resistance. Those with this allele acquire AD later in life than those with APOE 4. About 5–10% of people carry this allele. Disease resistance is connected to APOE 2. This variation delays AD development relative to APOE 4 bearers. This allele is seen in 5–10% of people. The most common variant of APOE, APOE 3, may not affect AD risk. Some groups with APOE 4 have a higher risk of AD and an earlier onset. About 15–25% of people carry this genotype, and 2–5% have two copies.

Multiple genes raise Alzheimer’s risk. Original risk gene APOE-e4 remains most important. Scientists believe 40–65% of Alzheimer’s patients have APOE-e4. One of three main APOE gene types is APOE-e4. Other two are APOE-e2 and e3. We inherit our parents’ APOE alleles. One copy of APOE-e4 from each parent increases Alzheimer’s risk. Two copies from each parent are riskier but not guaranteed. APOE-e4 increases vulnerability and symptoms earlier. About 2% of Americans have two APOE-e4 copies, while 20–30% have one. The rare AD-causing genes and the common APOE-e4 gene can be evaluated genetically. A person should not undergo routine genetic testing for AD risk until they have had proper counseling and understand the social and economical implications, according to the Alzheimer’s Association. Alzheimer’s patients may want to discuss genetic testing with their doctors because it may affect therapy. Anti-amyloid medications like aducanumab may cause more serious side effects in APOE-e4 carriers. Genetic counselors should be consulted before and after testing.

### 3.4 Differentiating genetic and extrinsic factors in AD

The term “extrinsic factors” refers to any external effect. The use of certain medications at the same time (drug–drug interaction), consuming alcohol, smoking, being malnourished, lacking access to clean water, and other environmental factors are all examples (6). Red meat delivers iron to prevent anemia. Excess iron in the body creates free radicals that damage cells. Iron accumulation in gray matter makes it sensitive to cognitive damage. AGE foods raise blood sugar. Due to improper glucose metabolism, senior Alzheimer’s patients’ brains have greater glucose levels, according to the National Institute on Aging. Brain neurons can be attacked by glucose without insulin. High glucose levels promote inflammation and cell damage, say researchers.

## 4 Environmental risk factors

### 4.1 Introduction to extrinsic factors

**Figure 1:**
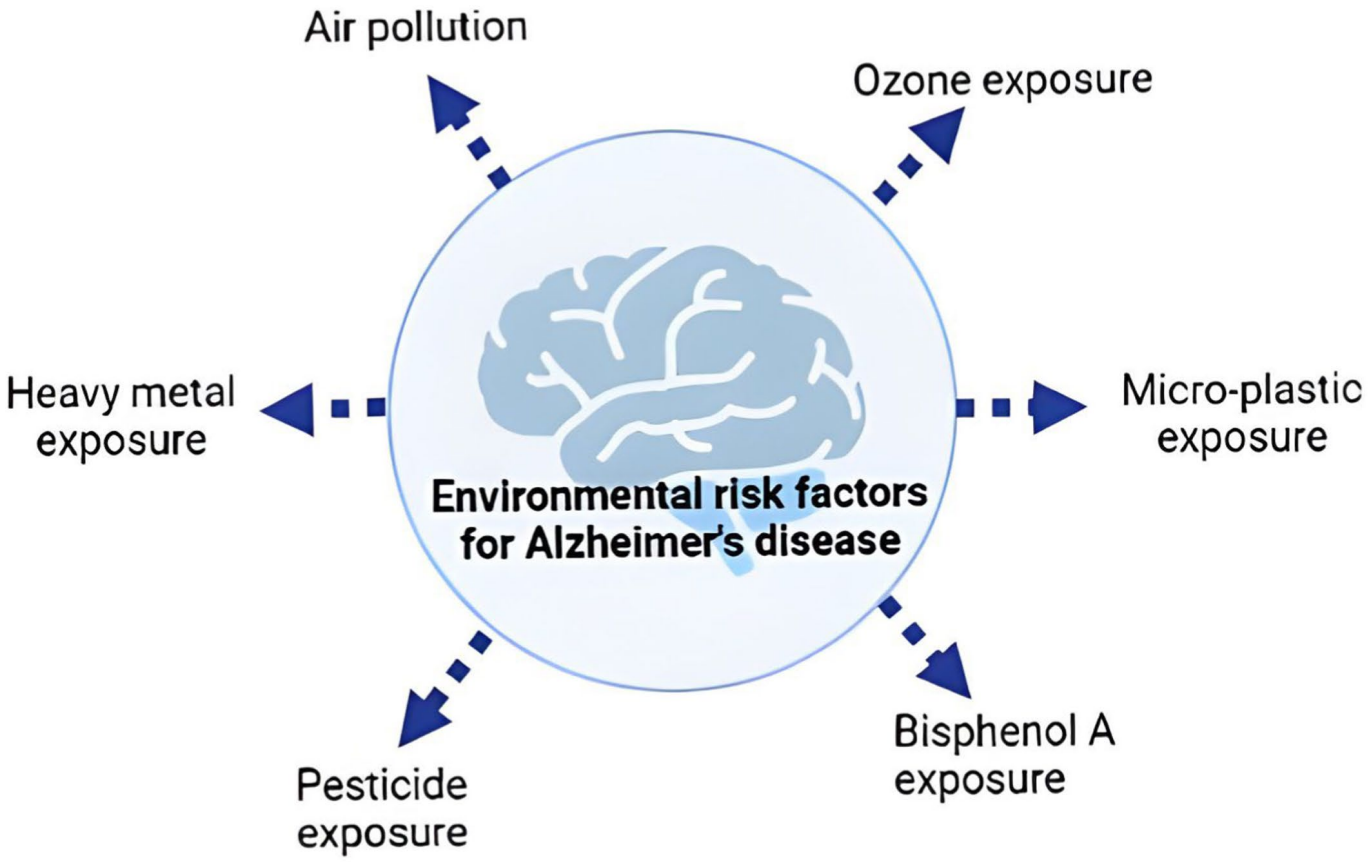
Common environmental risk factors of Alzheimer’s disease.

Environmental risk factors for dementia commonly share traits that impede brain impact assessment (Figure 1). These factors may share oxidative stress or inflammation, which will be explored in depth below. Many genetic and environmental risk factors overlap with dementia pathogenesis. This shows how complicated the sickness is. We also know little about how familial-EOAD and sporadic-LOAD reach the same neurological outcome (7). Previous studies have shown many AD risk variables that may seem unrelated. To explain AD’s intricacy, experts have presented several risk factor-based explanations. Based on epidemiological data, Henderson et al. presented two theories. The first explanation suggests that harmful or infectious particles accumulate or cause AD over time. The second idea suggests that environmental exposure, genetic predisposition, and/or an age-related increase in a biological mechanism necessary for brain function may cause AD. Genetic and environmental factors boosted a general aging brain process, demonstrating they are unlikely to directly affect it. The idea indicates that several AD risk factors release oxygen-free radicals, which grow with age and accelerate normal aging, causing AD (8).

To explain AD, Lahiri and Maloney (9) suggested the “dual hit” model. According to this idea, environmental stressors might activate dormant genes during early development. As the first “hit,” environmental stress affects gene regulation through epigenetic alterations such DNA methylation at promoter gene locations. Later in life, additional stressors modify gene expression for the second “hit.” The approach emphasizes the role of genes and environmental stress, which may include unrelated risk factors, in AD onset. Third theories emphasize the body’s “allostatic load” (8). Aging and anatomical system degeneration are the key risk factors for synapse loss, according to this idea. Stress or allostatic load during a lifetime influences early-onset aging. Stress causes hormonal changes in the brain, which can lead to hypertension, glucose intolerance, cardiovascular disease (CVD), and immune system dysregulation, influencing mortality (8). These processes cause the progressive degeneration of neurons, the gradual disconnection of synapses, and a rise in the activation of genes that produce reactive by-products such Aβ and tau.

### 4.2 Lifestyle factors

#### 4.2.1 Diet and nutrition

A high-fat, high-glycemic-load, high-cholesterol, or Western diet increases peptide accumulation and other neurodegenerative indicators in AD. In contrast, a DASH, Mediterranean, or low-fat diet protects against AD. Reducing oxidative stress, inflammation, and A peptide accumulation are key. Those that rigorously followed the Mediterranean diet had lower IL-6, TNF-α, CRP, and LDL. DASH emphasizes fruits, vegetables, and whole grains and limits red and processed meats. Sodium and saturated fat are low, but potassium, calcium, magnesium, and fiber are abundant. Both the Mediterranean and DASH diets have anti-inflammatory and oxidative stress-lowering qualities that prevent AD (10–12). Menus should emphasize vegetables, fruits, whole grains, low-fat dairy, and lean protein. Limit saturated fat and cholesterol-rich foods. Health requires some fats, but not all. Butter, solid shortening, lard, and fatty meats should be limited for heart health. Protein-calorie shortage is linked to cognitive decline and AD. Quality of life, cognitive function, and psychological and nutritional condition were all found to be considerably improved with sufficient dietary care for senior AD patients by Wu et al. (13). Improved sleep quality, as measured by the Pittsburgh Sleep Quality Index (PSQI), was also linked to dietary supplementation (13, 14).

#### 4.2.2 Physical activity and exercise

Regular exercise reduces dementia risk by 30%, according to research. Alzheimer’s risk halved. Aerobic multicomponent training with power and balance exercises enhances executive function, attention, processing speed, episodic memory, and procedural memory, among other health benefits. Through the production of antioxidant enzymes and growth factors like superoxide dismutase, eNOS, BDNF, nerve growth factors, insulin-like growth factors, and vascular endothelial growth factor, moderate and high intensity exercise improves cerebral blood flow, hyperemia, cerebrovasculature, and tau pathology. Many people, especially those at risk for or with AD or taking medication, may ignore physical activity, even though extensive studies are needed to understand how it provides therapeutic benefits to develop standard procedures (15).

#### 4.2.3 Sleep patterns and sleep disorders

Sleep problems are an Alzheimer’s symptom. Wakefulness at night is linked to AD. Brain wave studies suggest reduced dreaming and non-dreaming sleep. Melatonin production can be affected by dementia. This promotes sleepiness at night. As dementia progresses, brains produce less melatonin, making sleep difficult. Sleep disturbances are connected to cognitive deterioration and AD. Nighttime awakenings may cause Alzheimer’s. Sleep–wake rhythms alter CSF amyloid- and tau levels (16). Future cognitive decline and AD pathology are connected to disrupted sleep. Over 2 years, older women who slept 5 h/night had lower cognitive function than those who slept 7 h/night. Amyloid-positive cognitively normal older adults had considerably lower sleep efficiency (total sleep time/time in bed) than amyloid-negative participants, according to another study. Sleep metrics like sleep efficiency differ between asymptomatic AD patients and those without AD, raising the question of which came first: sleep disturbance or AD illness. Researchers have struggled to correlate sleep loss to AD (17, 18).

### 4.3 Environmental toxins

#### 4.3.1 Air pollution

Emerging animal model evidence suggests ignored risk factor. The US National Ambient Air Quality Standards regulate lead, sulfur dioxide, ozone, nitrogen dioxide (NO2), carbon monoxide, and particulate matter. Automobiles and industrial boilers produce CO, while motor vehicles, construction machines, power plants, and industrial boilers produce NO2. Ozone is formed from nitrogen oxides (NO2), volatile organic compounds, oxygen, and heat or light. The main sources of O3 are industry, power utilities, and cars (19). When waste products comprising this metal are burned, lead is emitted. Burning fuels containing sulfur also results in the production of sulfur dioxide (SO_2_), particularly in industrial settings.

The equivalent aerodynamic diameter of PM governs its settling velocity. PM10 refers to particles under 10 μm in diameter, whereas PM2.5 refers to particles under 2.5 μm. PM with a diameter of 2.5 to 10 μm is deemed “coarse,” whereas PM with a diameter below 2.5 μm is termed “fine.” Ultrafine PM (UFPM or PM0.1) with a diameter of less than 100 nm is important for neurotoxicity. Remember that PM10 encompasses coarse, fine, and ultrafine particles with a diameter of less than 10 m. The US Environmental Protection Agency (EPA) monitors ambient air pollution. PM0.1 is rarely monitored, whereas PM2.5 and PM10 are. To clarify, current rules consider particulate matter’s mass, which may underestimate UFPM’s contribution to PM. This component deserves major study due to the growing evidence of UFPM neurotoxicity and its relationship with neurodevelopmental and neurodegenerative diseases (20). Future PM0.1 regulation should consider alternatives to mass-based methods. Alternatives include particle number-based approaches. Instead of focusing on particulate matter mass, the number of PM0.1 particles would be considered. Such a technique would improve understanding of ultrafine particle health hazards and enable targeted regulatory interventions.

A rising corpus of epidemiological data suggests that ambient air pollution may impair the brain, accelerate cognitive aging, and increase the risk of AD and other dementias. Systemic and brain inflammation and AD symptoms are linked to early air pollution exposure. Furthermore, AD affects women more. Numerous studies show that moderate cognitive impairment is more common in men (21). SAD reasons are unknown in many patients. Recent research suggest that lead, chemical pesticides, and airborne pollutants may contribute to AD development. These findings suggest environmental factors may cause AD (22).

**Figure 2:**
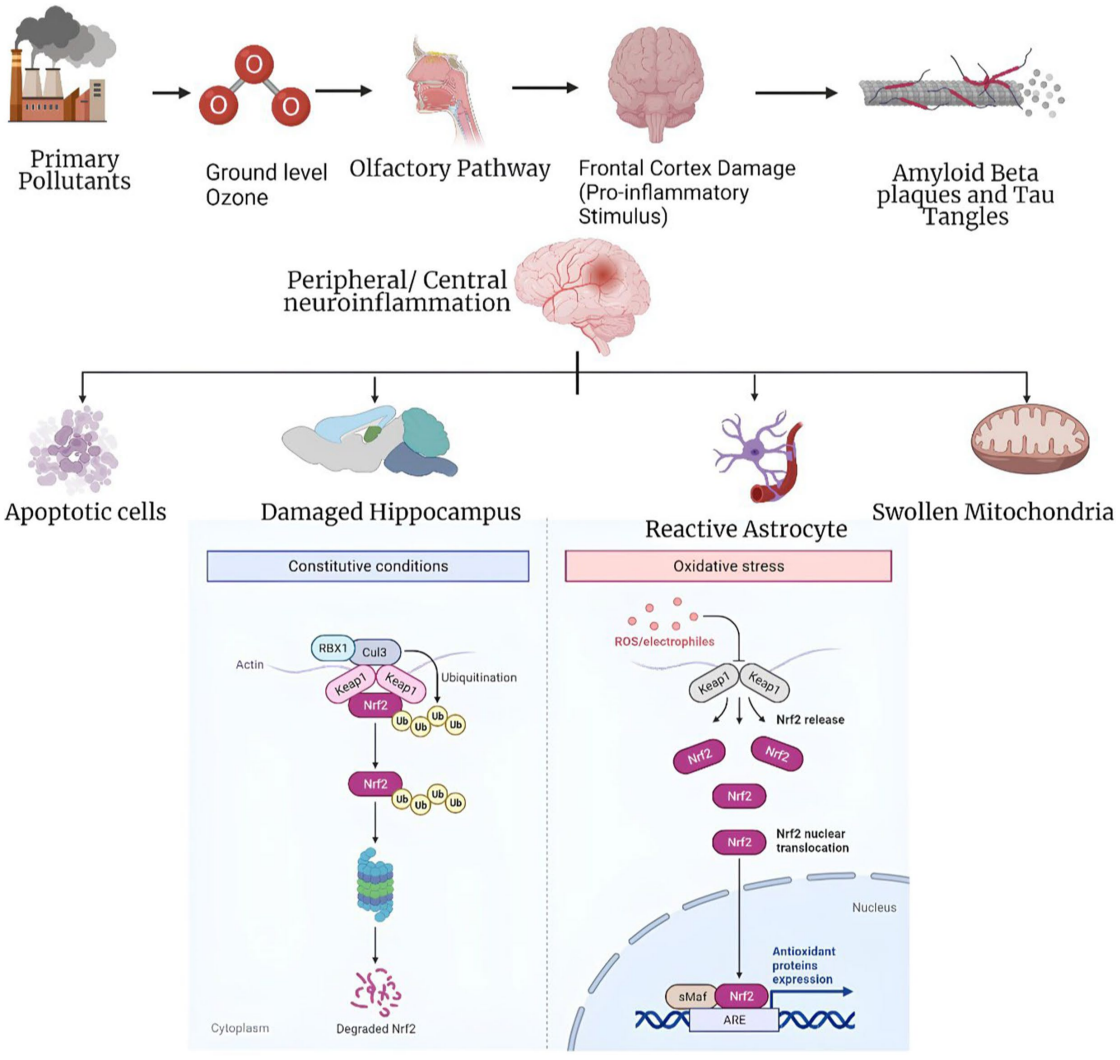
Panoptic view of the mechanism of ozone exposure involved in Alzheimer’s disease (23).

Ozone pollution may be a risk factor for neurodegenerative diseases, along with repeated air pollution exposure, concurrent illnesses like inflammatory disorders or type 2 diabetes, and genetic predispositions. Chronic low-dose ozone pollution mimics most of AD’s pathophysiology. This suggests a link between ozone pollution and AD-like pathology (Figure 2), highlighting the complex interplay between environmental factors and neurodegenerative diseases (24). Ozone may have health benefits, yet it may impair the nervous system. Ozone may benefit health in certain situations but harm neurological function in others. Consider both the pros and negatives of ozone exposure, especially when examining its effects on the nervous system. Ozone is a common urban contaminant and reactive oxidant. It is mostly generated in the troposphere by nitrogen oxides and volatile organic chemicals from fossil fuel combustion in sunlight. Thus, ozone levels rise throughout the day and fall at night. Secondary pollutants like tropospheric ozone harm humans and the environment. High ozone and nitrogen oxide levels harm climate, human health, the environment, and plants (25). The hypothesis suggesting the formation of ozone or ozone-like oxidants through the reaction of singlet oxygen with amino acids and their oxidation products gains support from the observation that methionine sulfoxide reacts with singlet oxygen to generate ozone or a similar oxidant (26).

To reduce misclassification and implement effective air quality initiatives, artificial neural network models like the Multilayer Perceptron have been developed. Ozone events are caused by causes that these models appropriately identify. These tools help us understand ozone generation mechanisms and build tailored ozone pollution mitigation solutions by using modern computational methods (27). Ozone affects the body in two ways. It first affects the olfactory bulb and nasal epithelium, then other brain regions. Second, when the lungs’ antioxidant capacity is exceeded, systemic oxidative stress affects various organs, including the brain. These dual pathways suggest that ozone may affect physiological systems outside the respiratory system (28). Ozone affects the body in two ways. It first affects the olfactory bulb and nasal epithelium, then other brain regions. Second, when the lungs’ antioxidant capacity is exceeded, systemic oxidative stress affects various organs, including the brain. These dual pathways suggest that ozone may affect physiological systems outside the respiratory system (29). Scientific studies have shown that urban youngsters without clinical health conditions who are exposed to fine particle matter and ozone levels above the US Environmental Protection Agency’s standards have reduced cognitive performance. Gender, BMI, and APOE status affect this impairment. These findings emphasize the influence of environmental contaminants on children’s cognitive capacities and the potential significance of individual variables in regulating this effect (30).

#### 4.3.2 Heavy metals

The prevalence of chronic metal exposure has escalated due to rapid human activities, resulting in the release of excessive amounts of metals into the environment. This exposure includes hazardous metals like lead, aluminum Emerging evidence from animal models indicates um, and cadmium that upset the equilibrium of metals in cells and organisms, in addition to necessary metals like zinc, copper, iron, and manganese. These metals have exceptional stability, solubility in precipitation from the atmosphere, and the capacity to be absorbed. Due to the ubiquitous use of these metals in numerous industrial, agricultural, residential, and technical applications, human exposure to them has dramatically expanded. Paints, mining operations, runoff from agriculture, treated wood, construction debris, outdated infrastructure for water supply, automobile emissions, fertilizers, batteries made from lead, and microplastics are some of the sources of metal pollution. Humans are primarily exposed to metals through ingestion, inhalation, and dermal contact. Efforts to mitigate the risks associated with metal exposure necessitate a thorough understanding of their sources, routes of exposure, and potential health effects (31, 32). Even trace amounts of non-essential metals can have profound toxic effects as they unintentionally interfere with the normal functioning of essential metals. Among the metals of utmost concern for public health, cadmium, lead, and aluminum exhibit high toxicity. These metals are recognized as systemic toxicants capable of causing damage to multiple organs, even at low levels of exposure. Importantly, there is evidence suggesting that the disruption of essential metal homeostasis and exposure to non-essential metals play a significant role in the development of AD.

In addition to its vital role in maintaining oxygen balance, iron plays a crucial role in coordinating a wide array of cellular functions. Among these include signaling pathways, DNA synthesis and repair, energy metabolism, respiration, and energy metabolism (33). Iron plays diverse roles in the CNS, including its involvement in important processes such as myelination of the spinal cord and white matter, as well as the synthesis, packaging, uptake, and degradation of neurotransmitters. Interestingly, the same property that renders iron biologically essential–its ability to undergo oxidation–reduction reactions—also poses a potential risk. This is because iron can convert hydrogen peroxide into highly toxic hydroxyl free radicals, leading to oxidative damage in the brain. Disruptions in iron homeostasis within the brain have been associated with various diseases. Low amounts of iron in the brain have been associated with undesirable pregnancy outcomes and cognitive developmental problems in children, including attention deficit hyperactivity disorder (ADHD) (34). Moreover, excessive accumulation of iron in adults has been associated with the development of neurodegenerative diseases, notably AD (35). As early as the moderate cognitive impairment stage of AD, changes in the iron levels in the brain can be seen. As a result, scientists have looked at the possible use of iron-based magnetic resonance imaging contrast, which can identify the amount of iron in the brain, as a biomarker for pathological changes associated with AD. Iron has a negative effect on AD mostly because of its cytotoxic redox characteristics, which become worse as the illness worsens. A peptide injection and genetic mitochondrial ferritin deletion led to an aggravation of neurological impairment in a mouse model, which was characterized by intracellular iron build-up and increased levels of oxidative stress (36).

It has been demonstrated through *in vitro* research that when Aβ aggregates, its affinity for iron rises, amplifying its neurotoxic effects (37). Due to the binding contact between iron and Aβ, the redox potential is altered in a way that encourages the redox cycling of iron, resulting in this occurrence. As a result, oxidative species are generated, and essential oxygen and biological reductants become depleted. Aβ’s binding to iron also leads to competition with other crucial iron-containing proteins. Notably, Aβ exhibits a significantly higher affinity for iron compared to transferrin by approximately eight orders of magnitude. Consequently, the accumulation of Aβ can disrupt iron homeostasis (38). Consistent with this concept, studies have demonstrated that the deposition of Aβ in the APP/PS1 mouse model is accompanied by alterations in iron-related proteins, namely, divalent metal transporter 1 (DMT1) and ferroportin 1 (FPN1). Specifically, the levels of DMT1 in the brain are observed to increase while the levels of FPN1 are reduced (39). After CNS damage, iron builds up in macrophages and microglia, which causes a change in cell activity, favoring a pro-inflammatory phenotype and lower phagocytosis efficiency. These investigations suggest a possible positive feedback loop between severe pro-inflammation and iron build-up. However, the specific biological mechanism by which iron affects glial cell activity is yet unknown.

Lead is a well-known neurotoxic substance that can cause nonspecific disruption in the brain. Its effects involve the induction of oxidative stress, endoplasmic reticulum stress, neuroinflammation, apoptosis, epigenetic changes, excitotoxicity, and disruption of essential metals in the brain. Animal models treated with lead have exhibited mechanisms and symptoms similar to those observed in AD. However, the effects of lead can vary depending on the species, timing, dose, and duration of exposure, although impairments associated with AD are consistently observed. Studies conducted on mice and rats have shed light on the susceptibility to lead exposure and its molecular targets. After CNS damage, iron builds up in macrophages and microglia, which causes a change in cell activity, favoring a pro-inflammatory phenotype and lower phagocytosis efficiency. These investigations suggest a possible positive feedback loop between severe pro-inflammation and iron build-up, although the specific biological mechanism by which iron affects glial cell activity is yet unknown. In contrast, adult animals treated with lead did not exhibit comparable consequences. Additionally, the developmental timing of lead exposure in mice influenced their performance in the Morris water maze, a test of learning and memory. Overall, lead exposure in animal models provides insights into the impact of lead on AD-related mechanisms and behaviors. These studies highlight the importance of considering factors such as timing and duration of exposure when examining the effects of lead on brain function (40).

Lead acetate was injected intraperitoneally into male rats between the ages of 8 and 9 weeks, and this caused the levels of A1-40 in the choroid plexus to rise by three times. Reduced RNA and protein levels of low-density lipoprotein receptor-1 (LDLR-1) were also linked to this lead therapy (23). A unique long-term lead exposure paradigm in monkeys has shown persuasive evidence connecting lead exposure to AD-related neurodegeneration. Female *Macaca fascicularis* monkeys were subjected to lead acetate exposure at a dosage of 1.5 mg/kg/day from postnatal day 1 to day 400. Upon reaching the age of 23, these aging primates exposed to lead exhibited elevated expression of amyloid precursor protein (APP) and amyloid-β (Aβ), along with augmented pathological neurodegeneration (41). Within the same cohort, it was observed that early-life exposure to lead resulted in increased levels of tau messenger RNA (mRNA), tau protein, as well as the transcriptional regulators of tau (Sp1 and Sp3). Additionally, specific sites on tau protein exhibited heightened phosphorylation, indicative of tau hyperphosphorylation (40). Early-life exposure to lead exerts a delayed impact on molecular pathways associated with AD during later stages of life.

Cadmium lacks essential physiological functions in humans and has been classified as a Group-I carcinogen by the International Agency for Research on Cancer (IARC). Various tissues actively absorb cadmium, which then enters the circulation. In adults, only minimal quantities of cadmium can normally traverse the blood–brain barrier (BBB). The blood-cerebrospinal fluid barrier, which includes the choroid plexus, prevents blood-borne poisons from entering the cerebrospinal fluid and preserves the homeostatic environment inside the central nervous system. Notably, the choroid plexus represents a major site of cadmium accumulation within the brain. Additionally, the olfactory nervous system may serve as a direct pathway for cadmium transport to the brain, thereby circumventing the BBB (42, 43). Mice exposed to cadmium exhibited impaired performance in tasks involving hippocampus-dependent spatial learning and memory, as well as olfactory memory. These cognitive deficits indicate an adverse effect of cadmium exposure on the functioning of the hippocampus and olfactory system, which are critical for spatial navigation and odor recognition, respectively (44). Cadmium has the ability to traverse directly into the central nervous system via the olfactory system, leading to long-lasting and irreversible damage. One of the detrimental effects of cadmium exposure is the inhibition of adult neurogenesis in both the hippocampus and olfactory bulb.

Animal studies have provided evidence supporting the biological associations between cadmium exposure and the aggregation of Aβ (amyloid-beta) as well as the accumulation of tau neurofibrillary tangles, both hallmark features of neurodegenerative diseases such as Alzheimer’s (45). Mice exposed to cadmium displayed impaired cognitive functions and memory deficits, accompanied by the presence of senile plaques in the brain. The suppression of α-secretase and the stimulation of amyloid-beta precursor protein (APP) processing through the α-secretase pathway may be the cause of the decline in learning and memory capacities shown in cadmium-treated mice. This altered AβPP metabolism leads to the accumulation of Aβ1–42 and the subsequent deposition of senile plaques. These findings suggest a mechanistic link between cadmium exposure, disrupted AβPP processing, and the development of neurodegenerative pathology associated with cognitive impairment (46). Exposure to cadmium has been shown to elevate the production of amyloid-beta (Aβ) and induce the formation of tau tangles, which are pathological hallmarks of AD. Furthermore, cadmium has the potential to affect cholinergic neurons, providing an additional pathway linking cadmium exposure to AD. Particularly, exposure to cadmium has been linked to an increase in cholinergic neuron cell death, altering AChE activity and causing basal forebrain cholinergic neurons to degenerate. These findings suggest that cadmium-induced toxicity on cholinergic neurons may contribute to the development and progression of AD pathology (47).

The most prevalent metallic element is aluminum, which ranks third among the materials that make up the Earth’s crust in terms of quantity. Due to its widespread industrial use, aluminum is incorporated into numerous consumer products that are widely accessible, such as drinking water, processed foods, infant formulae, cosmetics, toothpaste, antiperspirants, and various medical preparations and medications. Studies conducted on rodents, specifically rats and mice, have demonstrated that aluminum has the propensity to accumulate in specific regions of the brain, including the cortex, hippocampus, and cerebellum (48), after either parenteral or oral exposures. Numerous studies have investigated the levels of aluminum in the brains of individuals with AD and those without cognitive impairment, serving as controls. These investigations have consistently identified the hippocampus and the amygdala as the prominent brain regions where aluminum accumulates. Furthermore, several research findings have indicated that aluminum exposure can stimulate the expression of APP, which is the precursor of the Aβ protein. Additionally, aluminum has been shown to elevate the levels of Aβ-40 and Aβ-42 fragments within the brain and enhance the aggregation of Aβ protein (49). Moreover, scientific evidence has demonstrated that aluminum exposure contributes to the phosphorylation and aggregation of proteins, particularly the tau protein. Aluminum has been shown to promote the phosphorylation of tau protein, leading to the formation of abnormal aggregates. These phosphorylated tau aggregates are a hallmark of neurodegenerative diseases such as AD.

The detrimental effects of aluminum on tau phosphorylation and aggregation provide insights into its potential role in the pathogenesis of these neurodegenerative disorders (50). Furthermore, chronic oral administration of high doses of aluminum has been associated with altered synaptic plasticity in the hippocampus of rats. Studies have revealed that prolonged exposure to elevated levels of aluminum can disrupt the normal functioning of synaptic connections, impairing the ability of neurons in the hippocampus to undergo plastic changes that are essential for learning and memory processes. These findings highlight the potential impact of aluminum on synaptic function and provide insights into the mechanisms underlying its detrimental effects on cognitive function (51). Numerous studies have provided evidence that aluminum can disrupt the cholinergic system, which is known to be involved in the pathogenesis of AD. Both *invivo* and *in vitro* investigations have consistently demonstrated alterations in the activity of AChE, a key enzyme involved in the breakdown of acetylcholine. Additionally, studies have revealed changes in acetylcholine-mediated neurotransmission in response to aluminum exposure. These findings suggest that aluminum -induced disruption of the cholinergic system may contribute to the pathophysiology of AD (51, 52).

Manganese (Mn) is an essential trace metal that plays a crucial role in various neurological processes and brain development. The dysregulation of manganese levels has been observed in a wide range of neurological disorders, highlighting its significance in maintaining normal brain function. Early evidence of manganese-induced brain toxicity emerged from observations of acquired manganism in miners and individuals misusing drugs. The discovery of inherited manganese transportopathies, which lead to neurodevelopmental and neurodegenerative syndromes, further confirms the neurotoxic potential of this element. The brain maintains tight regulation of manganese homeostasis through cellular and systemic mechanisms to support enzymatic processes that rely on manganese. Manganese can cross the blood–brain barrier via the choroid plexus, the olfactory epithelium, olfactory neurons, and direct intra-axonal uptake at presynaptic nerve ends. In addition to its essential role in normal neuronal function, manganese also provides neuroprotection. It acts as a critical cofactor for astrocytic glutamine synthetase, a key enzyme involved in regulating the glutamate-glutamine and GABA-glutamine cycles, as well as facilitating normal synaptic function and excitatory glutamate turnover (53–55).

Manganese has the potential to interfere with normal protein processing and clearance mechanisms in the brain, which can result in the accumulation of abnormal protein aggregates. One specific example is the increased production and deposition of beta-amyloid protein, known for its association with the formation of amyloid plaques in AD. Manganese exposure has been linked to the disruption of mitochondrial function, which plays a critical role in cellular energy production and metabolism. Impaired mitochondrial function can lead to energy deficits within cells and the generation of harmful byproducts that contribute to neurodegeneration. It is important to acknowledge that while these mechanisms shed light on the potential role of manganese in AD, the precise processes and interactions involved are still under investigation (56). Given the pervasiveness and global frequency of heavy metal exposure, even small increases in the chances of AD and related dementias would have a significant influence on disease burden at the population level. The hazards of AD and other dementias that may be brought on by heavy metal exposure must thus be addressed through exposure control measures. These measures should be implemented to effectively reduce the potential adverse effects of heavy metal exposure on cognitive health.

#### 4.3.3 Pesticides

A pesticide is defined as a substance or combination of substances designed to prevent, eradicate, or control pests, which can include both chemical and biological agents (57). Different classes of pesticides exist, each with specific targets and mechanisms of action. Among the most notable classes are organophosphates (OPs), pyrethroids, carbamates, organochlorines, and neonicotinoids. These classes represent a diverse range of pesticides commonly used for various pest control purposes muir (58). Individual pesticides have different solubility properties, which can have a big impact on how long they last in the environment and how likely they are to bioaccumulate. Pesticides that are particularly water soluble, like the widely recognized herbicide 2,4-D, tend to break down more quickly and are less prone to build up in organisms. Contrarily, lipid-soluble pesticides from the organochlorine family, like DDT and DDE, have a higher propensity for bioaccumulation. There is little knowledge about how the central nervous system (CNS) detoxifies pesticides, and the CNS detoxifying systems show significant polymorphism, which causes significant inter-individual variation in vulnerability to the neurotoxic consequences of insecticides. Furthermore, further research is necessary to fully understand the long-term consequences of pesticide exposure on brain development (59).

Pesticide molecules, characterized by their small size and lipophilic nature, have the ability to cross the blood–brain barrier and enter various components of the CNS, including neurons, brain microvessels, and glial cells. These pesticides target and interfere with BBB receptors in the CNS, increasing chronic toxicity and interfering with receptor-mediated transcytosis. They also obstruct the delivery of drugs to the brain tissue through endothelial cells of the brain. This can result in reduced drug efficacy and other adverse effects. OPs, which are a subset of a vast family of chlorinated hydrocarbon compounds, have been extensively utilized in agricultural practices and public health initiatives, posing serious environmental and health risks. These substances have spread across the environment and have a propensity to bioaccumulate. Humans are at the highest levels of the food chain, making them particularly vulnerable to health impacts from ingesting harmful substances, which happen in much larger quantities thanks to a process called biomagnification. OPs have high lipophilicity, sluggish environmental degradation, and a propensity to build up in fatty tissues and the blood. They are frequently linked to the development of neurodegenerative disorders (60).

Both OPs and carbamates are known to block the enzyme acetylcholinesterase (AChE), which causes a build-up of acetylcholine (ACh) in the synaptic clefts. This is how both chemicals work. Although the acute toxicity symptoms brought on by carbamate and OP poisoning may be comparable, carbamate poisoning often has a quicker recovery time than OP poisoning. On the other hand, OPs have been linked to a greater prevalence of long-term side effects, including developmental neurological damage, cognitive sequelae, and organophosphate-induced delayed polyneuropathy (OPIDP) (61). The fundamental mode of action for most OPs involves disrupting the transmission of nerve signals across synapses or from nerves to muscle fibers. Various pesticides achieve this by various means; however, the ultimate outcome is a disruption in the normal propagation of nerve signals. OPs primarily exert their effects by modifying the movement of ions across the membranes of nerve cells, thereby altering the nerve’s ability to generate electrical impulses.

OPs have a similar neurotoxic function, but the phenotypic effects of exposure to them can differ greatly. Symptoms may appear after acute or chronic exposure. AChE is inhibited during acute cholinergic syndrome (ACS), the first stage of OP poisoning. ACS, which affects the nicotinic and muscarinic receptors, can appear soon after OP exposure. Reflecting the varied effects of OPs on both CNS and peripheral nervous system (PNS) pathways, the symptoms of OP exposure are complex and go beyond localized locations. The intermediate syndrome (IMS), which may appear 1–4 days after ACS, is characterized by the development of muscular weakness, especially in the neck, respiratory system, and proximal limbs. Around 14 to 21 days after acute exposure, peripheral muscular weakening becomes visible if untreated. Only 20% of people exposed to OPs are predicted to advance from the ACS stage to the IMS stage (62). The IMS is frequently accompanied by failure of the respiratory system due to the paralysis of nicotinic receptors. OPs can interact with secondary hydroxyl sites on enzymes other than AChE, depending on the particular chemical structure. The condition known as organophosphate-induced delayed neuropathy (OPIDN) is another kind of toxicity that can result from exposure to OP pesticides. The onset of OPIDN typically occurs approximately 2–3 weeks after the acute cholinergic syndrome (ACS), with the severity and occurrence being influenced by the dose and specific chemical structure of the OP compound (63). OPIDN is characterized by the degeneration of distal axons in both the central nervous system (CNS) and PNS. Distal muscular weakness, coordination issues, diminished senses in the digits and feet, and other neurological deficits are all symptoms of this degenerative illness. Neuropathy target esterase, a crucial enzyme involved in the start of neurite development during neural morphogenesis, is inhibited as the primary mechanism causing OPIDN. Axons and dendrites grow out of neurites to become vital parts of the nervous system. It is interesting that OPIDN has negative effects only when NTE inhibition is at or above 70% of its activity (64).

Diazinon and chlorpyrifos have been shown to inhibit DNA synthesis in experiments employing brain cell lines. Both fetal development and postnatal behavior are significantly influenced by the timing of prenatal exposure to OPs. The impaired cognitive development at 2 and 6 months of the child has specifically been related to OP exposure during the first and second trimesters of pregnancy. Conversely, exposure to OP throughout the third trimester of pregnancy is linked to impaired motor and communicative development at 6 months of age (65). According to recent studies, continuous exposure to OPs among agricultural workers is linked to neurological anomalies, such as neurodegenerative diseases, attention difficulties, and deterioration of short-term memory (66).

OP pesticides have an impact on the cholinergic system, which has been directly connected to neurodegenerative diseases. ACh, one of the important neurotransmitters associated with brain cell signaling, is essential for memory-related functions. ACh in the basal forebrain is recognized to be crucial for memory and learning. Hence reduction in ACh levels is a critical element in disorders that cause memory loss, such as AD. Memory-related illnesses are impacted by the alteration of ACh equilibrium at neuronal junctions in the brain brought on by OP pesticides (67). Recognizing and investigating the correlation between OP exposure and neurodevelopmental outcomes and behavioral effects will promote advancements in the regulation and management of OPs in agricultural, industrial, and domestic settings globally. By addressing this relationship, it becomes possible to enhance practices and protocols for the safe and responsible use and handling of OPs, leading to a safer environment for individuals and minimizing the potential risks associated with these chemicals.

### 4.4 Psychological and social factors

#### 4.4.1 Stress and chronic stressors

The relationship between stress and dementia is complex. The immune system, which is known to play a significant role in the onset of dementia, is impacted by stress. One of the main stress hormones, cortisol, has been related to memory loss. Disorders like sadness and anxiety are also strongly linked to stress. In addition, these have been proposed as potential dementia risk factors (17). Dementia and stress may be connected in a number of ways. Dementia is linked to a compromised immune system, which is known to be impacted by stress. Cortisol, a major stress hormone, has been related to memory impairment. Conditions like sadness and anxiety are also strongly connected to stress. These too have been proposed as potential dementia risk factors. Stress appears to have a direct impact on some of the pathways behind dementia, according to recent study in animal models. Understanding whether any of these theories are true, however, has proven to be a lengthy and winding road, as is the case with many things in the academic world.

#### 4.4.2 Social isolation and loneliness

The current study illuminates the complex web of connections between social isolation and key cardiovascular disease risk factors. Our data reveal that, after controlling for effects of age and sex, a lack of social capital (loneliness and lack of social support) is consistently linked to standard AD related dementia (ADRD) risk variables. To the best of our knowledge, this is the first study to use data from two nationally representative population cohorts of older adults from two different countries to demonstrate a link between social isolation and a comprehensive array of the most studied risk factors of ADRD. Sleep is a sample example of the many measurements of personal habits and lifestyle characteristics that indicated multiple and substantial relationships with social isolation. Loneliness and isolation were strongly linked to sleep disruption (68, 69). Perceived social isolation has been linked by other researchers to increased anxiety and poorer sleep quality due to heightened attention for social dangers. Many studies corroborate this hypothesis by showing that those who report higher levels of stress are more likely to experience loneliness and less social support. It has been hypothesized that stress accumulation and emotional coping contribute to the underlying reasons why lonely persons are more likely to engage in risky behaviors including smoking, drinking excessively, and watching excessive amounts of television. The number of studies linking poor sleep, cigarette smoking, heavy drinking, and TV addiction to dementia and ADRD keeps expanding. Our findings across two large cohorts revealed substantial links between loneliness and lack of social support and these potentially modifiable lifestyle variables that affect the onset of dementia (69).

### 4.5 Infectious agents

#### 4.5.1 Viruses

Herpes simplex virus type 1 (HSV1) is one of the infections that has been linked to A- and tau-pathology. However, the causal connection is contested due to the existence of contradictory data.5 We postulated that soluble A counteracts the neurotoxic effects of HSV1. In AD, soluble Aβ levels are lowered. As a result, elevated HSV1 activity may contribute to neuronal dysfunction and cell death in AD. Using cell culture and animal models, we explore how human herpes simplex virus 1 (HSV1) causes amyloid-β (Aβ) buildup and neurofibrillary tangles of hyperphosphorylated tau (pTau) in AD. A seems to have a virostatic effect (70).

#### 4.5.2 Bacteria

In particular, previous research has shown that people with advanced Alzheimer’s have higher ratios of Bacteroidetes, which are primarily known for their ability to fight off other pathogens, to Firmicutes, which are associated with obesity and diabetes. The link between oral bacteria and AD is supported by modest evidence at present, but was robust when oral bacteria were detectable in the brain. Additional data is required to elucidate the connection between certain bacterial species and the emergence of AD (71).

#### 4.5.3 Fungi

The brains of AD patients frequently include fungal species such *Candida* spp., Malassezia spp., Cladosporium spp., and Alternaria spp. In immunocompromised patients, these fungi are a common source of infection because of their opportunistic nature. Fungal meningitis can be caused when these organisms travel to the brain. Systemic infection following epithelial barrier breakdown via skin or gut colonization is the most common route of entry. It has been hypothesized that Candida species, once within the brain, induces fungal glial granulomas with accumulating amyloid precursor protein (APP). Amyloid beta (A) can be created when APP is cleaved. By stimulating helper T-cell (Th) 1 and Th17 immunological responses, Malassezia species can cause neuroinflammation. Furthermore, it is unclear how Cladosporium species and Alternaria species contribute to the pathogenesis of AD, however this may be connected to neuroinflammation. The two types of fungi may have contributed to the development of acetylcholinesterase (AChE) inhibitors in the human brain. Neuroinflammation and neurodegeneration are both exacerbated by the simultaneous presence of these four fungus in the brain (72).

### 4.6 Review of epidemiological studies linking extrinsic factors to AD

Three primary etiological hypotheses of AD’s development have been proposed, based on epidemiological studies, genetic studies, neuroimaging technologies, and neuropathology research: hereditary, vascular, and psychosocial. The etiological role of additional factors, including as nutrition, occupational exposure to various chemicals, and inflammation, is currently unsupported by research. Genetic variables are the most crucial for early diagnosis and the implementation of primary or secondary prevention measures (73).

## 5 Mechanisms of extrinsic factors in AD development

### 5.1 Neuroinflammation and neurodegeneration

The pathophysiology of AD is increasingly thought to involve interactions between neuronal and immune systems in the brain. Through their interactions with pattern recognition receptors on micro- and astroglia, misfolded and aggregated proteins initiate an innate immune response, which is characterized by the release of inflammatory mediators and contributes to the progression and severity of disease. Genes that raise the chance of developing sporadic AD appear to encode for components that control glial clearance of misfolded proteins and the inflammatory response, according to a genome-wide investigation. Disease progression is likely aided by environmental factors such as systemic inflammation and obesity, both of which disrupt the brain’s immunological functions. This review is an update on the state of our understanding, with an emphasis on the most recent and fascinating discoveries. Future preventative or therapeutic methods for AD are likely to involve modulating risk factors and intervening with the outlined immunological pathways (74).

#### 5.1.1 Microglial activation and neuroinflammation

Neuroinflammation and other disorders of the central nervous system are greatly impacted by microglia, the CNS’s immune cells. Activation and functional change of microglia have been hypothesized as crucial mediators in the development of persistent neurodegenerative disease after traumatic brain injury (75). Growing research indicates that activated microglia are a persistent source of several neurotoxic chemicals, such as tumor necrosis factor α, nitric oxide, interleukin-1, and reactive oxygen species, that cause gradual neuronal damage (75).

#### 5.1.2 Role of extrinsic factors in promoting neuroinflammation

Complex internal mechanisms and responses to external signals are required for the regeneration, repair, and regrowth of injured axons. Injured neurons in the central nervous system have a variable innate capacity to enhance axon development and elongate depending on a combination of intrinsic and extrinsic signals. Axonal growth and regeneration capacity declines with age due to changes in both intrinsic and extrinsic mechanisms inside neurons. Axon development following injury is influenced by numerous neuronal-intrinsic factors, many of which are involved in multiple pathways (PTEN/mTOR pathway). Mitochondrial function, neuronal viscosity and axonal transport, the cytokine signaling regulators Suppressor of Cytokine Signaling 3 (SOCS3) and Signal Transducer and Activator of Transcription 3 (STAT3), Wnt/Ryk, insulin-like growth factor (IGF)-1, the tumor suppressor p53, and Krüppel-like factors (KLFs). Our earlier review, which centered on how the intrinsic processes of neurons evolve with age, provides extensive coverage of these topics (76). Numerous essential cellular functions and responses to external stimuli are known to be mediated by mitogen activated protein kinases (MAPKs). Attractive targets in a variety of illness models are the p38 and c-Jun N-terminal kinase (JNK) signaling cascades, which have been linked to the regulation of transcription and translation of inflammatory mediators (77).

#### 5.1.3 Neurodegenerative processes and their relationship with extrinsic factors

Bone morphogenetic proteins (BMPs), Notch, and the Janus kinase/signal transducer and activator of transcription (JAK–STAT) pathway are examples of basic helix–loop–helix (bHLH) transcription factors and signaling pathways that regulate the transition from neurogenesis to gliogenesis during development. Extrinsic and intrinsic factors can interfere with differentiation in a variety of ways, including blocking differentiation in a particular lineage, switching to a different lineage, or blocking differentiation overall. Neurodevelopmental, neurodegenerative, and neuropsychiatric illnesses can all be traced back to aberrant differentiation, which is linked to defective brain development and neurological diseases. Increased neuronal density in the prefrontal cortex throughout childhood, for instance, is associated with autism spectrum disorder (ASD) (78).

### 5.2 Oxidative stress

Multiple neurodegenerative illnesses have implicated oxidative stress in their onset and/or progression. The purpose of this chapter is to provide a synopsis of the research connecting the pathophysiology of AD to the excessive production of reactive oxygen species. It was also shown that oxidative stress altered the body’s inflammatory reaction. The pathogenic processes of oxidative stress and neuroinflammation are distinct, but they are intertwined and mutually affective. Although much progress has been made in our understanding of the causes and progression of neurodegenerative illnesses, much more research is needed before effective treatments can be created (79, 80). The most prevalent enzymatic and non-enzymatic antioxidants have been highlighted in this paper as potential therapy alternatives for the disorders addressed in this article because of their ability to alter oxidative stress and slow down the symptoms of these neurodegenerative diseases. Neurodegenerative disorders are characterized by a diminished inflammatory response. Neuroinflammation and oxidative stress (OS) have been found to be intricately related in the pathophysiology of neurodegenerative illnesses, and as such, both are crucial factors that must be considered when analyzing their development and progression (81). OS is made by reactive species secreted by inflammatory cells. Increased expression of pro-inflammatory genes can be facilitated by certain reactive oxygen species (ROS) and reactive nitrogen species (RNS). Therefore, in the context of disease, neuroinflammation and OS can mutually promote one another. The inflammatory response is a protective mechanism when the body’s reduction and oxidation reactions (redox) are in equilibrium; but, in the case of neurodegenerative events, there is a redox imbalance. Therefore, the inflammatory response fails to properly function, causing central nervous system inflammation (82).

### 5.3 Vascular factors and blood–brain barrier dysfunction

Dementia and neuronal degeneration are linked to impairments in the blood–brain barrier (BBB) and oligemia (lower cerebral blood flow), which in turn are caused by vascular factors (stroke, hypertension, diabetes, etc.) and hereditary factors (such as APOE4). By contrast, the amyloid-beta (A)-independent route (blue) involves the collapse of the blood–brain barrier (BBB), the release of neurotoxins, and the development of oligemia due to a combination of vascular and genetic variables. The first BBB breach hinders A clearance and the amyloid precursor protein (APP), leading to A buildup in the brain, while the A-dependent pathway is active (green). Tau (p-tau) is phosphorylated by amyloid- (A) and vascular hypoperfusion, leading to the formation of neurofibrillary tangles (NFTs). Dementia (particularly AD) is the end result of neurodegeneration, synaptic dysfunction, and neuronal damage that both pathways generate (83, 84).

### 5.4 Neurotransmitter imbalances

Neurotransmitters, the chemical mediators responsible for transmitting impulses between brain cells, are depleted as brain cells die off. AD is associated with decreased levels of the neurotransmitter acetylcholine in the brain. The brain’s various regions gradually atrophy as we age. Remembering is typically the first thing to go when this happens. AD can manifest in a variety of unexpected ways, each affecting a unique part of the brain. Memory issues may not be the first to surface, but rather issues with vision or language. An imbalance of many neurotransmitters is seen in the brains of AD patients, and dopamine depletion is possible in brains with AD disease but preserved cognition (85). GABAergic neurotransmission also suffers severe pathogenic alterations in AD, and it may be a promising therapeutic target for this neurodegenerative condition, contrary to what was once thought (86).

### 5.5 Impaired autophagy and protein clearance

Autophagy flow may be reduced if substrate identification is impaired. Several neurodegenerative disorders, characterized by synaptic dysfunction and axonal degeneration, can be traced back to impaired autophagy. Induction of autophagy as a treatment option is plausible. Increases in ROS and cellular stress occur when the autophagy/mitophagy process is disrupted and ROS are not neutralized and mitochondria are not recycled. An further obstacle for the autophagy/mitophagy process is the formation of ROS, which can damage DNA, proteins, and mitochondria. Thus, the two paths that converge on selective motor neuron death are regulation of autophagy/mitophagy via mitochondrial-derived ROS and mitophagy-dependent mitochondrial quality control (87, 88).

### 5.6 Impact of extrinsic factors on beta-amyloid and tau pathology

For nearly 30 years, the amyloid hypothesis has been the prevailing theory in Alzheimer’s research. This hypothesis states that synaptic accumulation of the peptide amyloid- is the primary cause of AD pathogenesis. In light of the dismal results from clinical studies of anti-amyloid therapies, however, substantial doubts regarding its validity have recently emerged. Thus, the focus of AD studies has changed from amyloid to other entities such as microglia, astrocytes, apolipoprotein E, and others that may play a role in the disease’s etiology. While it is evident that all play a role, the precise nature of that role is often obscured by the pleiotropy of those roles. Here, we suggest that the local modulation of amyloid- levels is a common function shared by all of these AD-related entities and may be crucial to their role in AD pathogenesis. We also discuss the present understanding of amyloid-'s effects at the synapse in health and disease, focusing on the possibility of its interaction with tau, the key protein associated with AD. There is strong evidence in favor of the amyloid hypothesis, and the fact that a wide variety of cell types, chemicals, and processes are linked to AD may actually strengthen it (89). Neurons were confirmed to be present in culture by staining with antibodies against neuronal markers such as III-Tubulin, MAP2, Tau, and tetanus toxin, as well as the immature neuronal postmitotic marker internexin. Furthermore, electrophysiological investigations have demonstrated that these cells exhibit properties similar to those of early neurons. Hypoxia induces Aβ aggregation, tau hyperphosphorylation, BBB failure, and the disturbance of calcium homeostasis, all of which contribute to the progression of AD. Hippocampal neurogenesis has been found to be negatively regulated by Aβ aggregation and tau hyperphosphorylation in the past. Human neural progenitor cells and brain organoids were both susceptible to infection by SARS-CoV-2, and the infected organoids showed changes in tau, including hyperphosphorylation of tau at T231, neuronal cell death, and other abnormalities (90–93).

## 6 Interplay between genetics and extrinsic factors

### 6.1 Gene–environment interactions

Genetic epidemiology is a new field that focuses on understanding how genes and environments interact. However, epidemiologists have argued at length on the nature, source, and detection of interaction when taking into account the joint effects of risk factors in disease causation. The interaction between genes and the environment merits the same careful consideration as the interaction between any two independent risk variables. This review aims to provide a clear description of gene–environment interaction and to offer strategies for identifying it through research. The exact origin of AD remains unknown. Instead, it may be affected by a combination of genetic and non-genetic variables, including one’s environment and way of life. Because of this, a person’s genetic make-up may include both risk factors for and protection against AD (94).

### 6.2 Epigenetic modifications

Modifications to DNA methylation, histone modifications, and microRNA (miRNA) expression are examples of epigenetic modifications that alter gene expression independently of changes to DNA sequences (95). Epigenetic mechanisms are dysregulated in AD, leading to changes in gene expression at the transcriptional level (upregulation, downregulation, or silence) due to DNA hypermethylation, histone deacetylation, and a generally suppressed chromatin state. Therefore, these epigenetic mechanisms or their modulators have shown enormous promise as a therapeutic target in AD (96).

### 6.3 Susceptibility genes and extrinsic factor sensitivity

A higher risk of getting an illness due to one’s genetic makeup is called a genetic predisposition (genetic susceptibility). Certain variations in a person’s DNA can cause hereditary predispositions. The amyloid precursor protein (APP) gene, the presenilin 1 (PSEN1) gene, and the presenilin 2 (PSEN2) gene have all been linked to the early-onset familial variant of the illness. However, fewer than 5% of AD cases are caused by mutations in these genes. The remaining 95% of people with AD are sporadic late-onset instances, the etiology of which is complicated by interactions between environmental factors and individual genetic makeup. Early-onset familial AD is caused by mutations in three genes, all of which exhibit almost 100% penetrance with autosomal dominant inheritance. The amyloid precursor protein (APP), presenilin 1 (PSEN1), and presenilin 2 (PSEN2) are the names of the genes in question. It is well known that sporadic late-onset instances of AD are more likely to involve the apolipoprotein E (APOE) gene. Through the use of genetic linkage analysis on a cohort of families with late-onset AD, APOE was pinpointed as a disease locus due to its position in the highest linkage region on chromosome 19 (97).

#### 6.3.1 Summary of non-environmental risk factors in AD

✓ The interplay between genetic and environmental elements is the key in understanding dementia complexity, evident in models like the “dual hit” and “allostatic load.”
✓ Genetics are pivotal in early AD diagnosis and prevention strategies, environmental influences like systemic inflammation, impaired cellular mechanisms, and neurotransmitter imbalances worsen neurodegeneration.
✓ Dietary patterns play a crucial role, with high-fat, high-glycaemic load diets elevating Alzheimer’s markers while Mediterranean and DASH diets mitigate oxidative stress and inflammation, safeguarding against AD.
✓ Regular physical activity reduces dementia risk substantially and enhances cognitive functions by promoting growth factors and cerebral blood flow.
✓ Sleep disruptions are linked to Alzheimer’s pathology, affecting amyloid and tau levels, yet the relationship between sleep disturbances and the disease remains a subject of exploration.
✓ Lifestyle changes, risk reduction strategies, vaccinations, and early intervention through public health policies stand as potential avenues for AD prevention.

#### 6.3.2 Summary of environmental risk factors in AD

The role of environmental contaminants in the development of AD is an area of active research and investigation.

**Figure 3:**
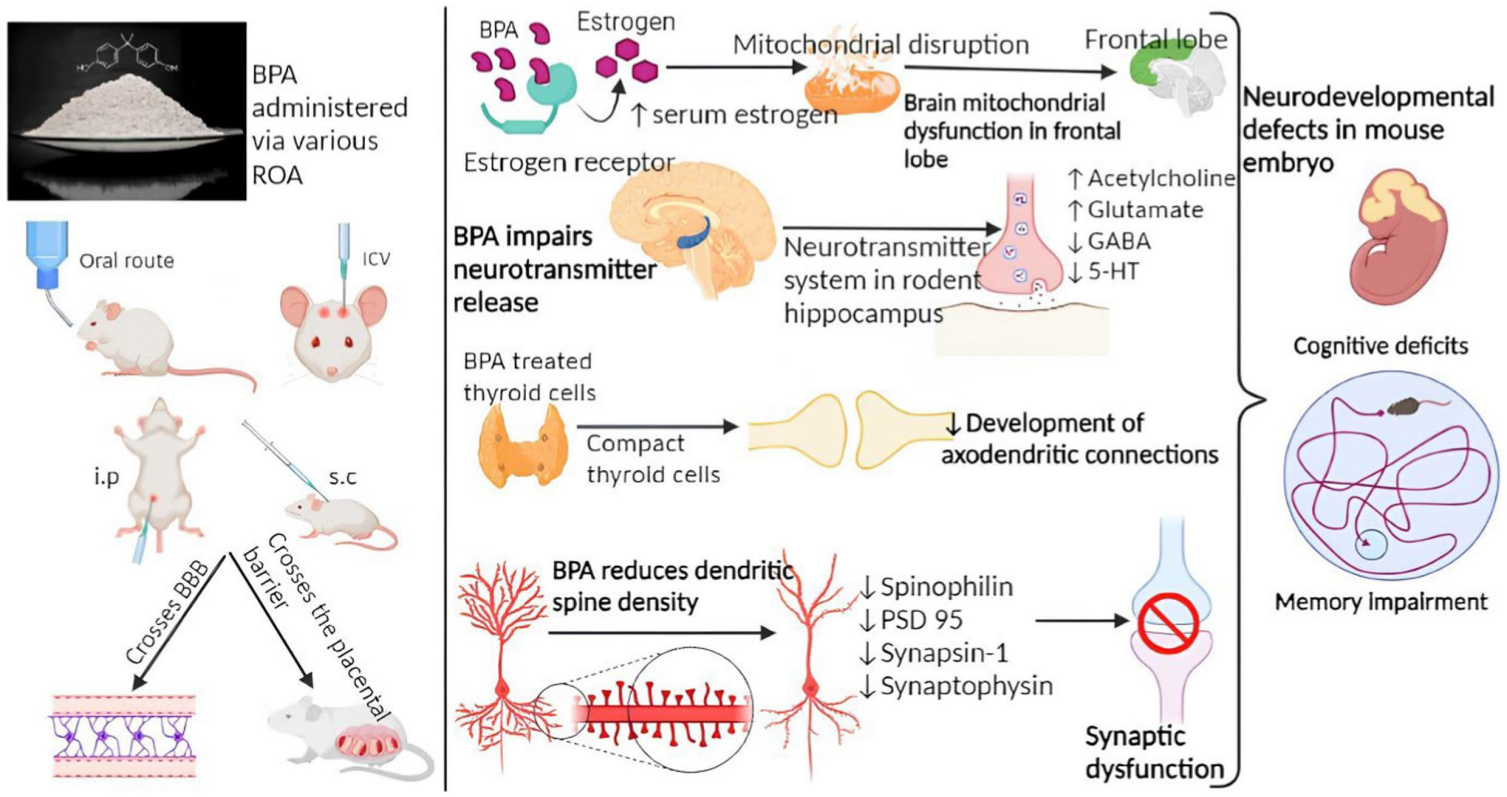
Mechanism of BPA involved in Alzheimer’s disease pathology (29).

✓ Air pollution: Studies have suggested a potential link between long-term exposure to air pollution, particularly fine particulate matter (PM2.5) and nitrogen dioxide (NO2), and an increased risk of cognitive decline and AD. Air pollution contains various harmful components, including heavy metals, organic compounds, and oxidative stress-inducing agents, which may trigger neuroinflammation, oxidative stress, and the accumulation of amyloid-beta plaques and neurofibrillary tangles, hallmarks of AD.
✓ Heavy metals: Certain heavy metals, such as lead, cadmium, manganese, and aluminum, have been investigated as potential contributors to AD. These metals can be present in tainted food, water, air, and workplace environments. Animal and *in vitro* studies have demonstrated that heavy metals can promote the accumulation of amyloid-beta plaques, induce oxidative stress, and cause neuroinflammation, which are all associated with Alzheimer’s pathology.
✓ Pesticides: Some studies have suggested a possible association between exposure to certain pesticides and an increased risk of AD. Pesticides, such as organophosphates and pyrethroids, have been investigated. Animal and cellular studies have shown that these chemicals can lead to neurotoxicity, mitochondrial dysfunction, oxidative stress, and neuroinflammation, which are mechanisms implicated in AD.
✓ Plastic: Studies have suggested that certain components of plastics, such as BPA, may contribute to the accumulation of beta-amyloid plaques, a hallmark of Suresh et al. (98). These studies have been conducted primarily in laboratory settings or on animal models (Figure 3). However, it’s important to note that the doses of plastic components used in these studies are often much higher than what humans typically encounter in real-life scenarios. Overall, the role of plastic exposure in the development of AD remains uncertain. The field of research is still evolving, and more studies are needed to better understand the potential mechanisms and establish definitive conclusions.

## 7 Prevention and intervention strategies

### 7.1 Lifestyle modifications

Maintaining a healthy weight can be achieved by a combination of balanced food and regular physical activity. Brain health, mental wellbeing, and emotional stability can all benefit from regular physical activity. Quitting smoking today can lower your chance of developing cardiovascular disease, cancer, lung disease, and other smoking-related disorders, and may help you keep your brain healthy. The likelihood of developing dementia increases with age, but there are steps you may do to mitigate this risk. Some examples are going for regular walks, eating right, and challenging your brain (Figure 3).

### 7.2 Environmental risk reduction

**Figure 4:**
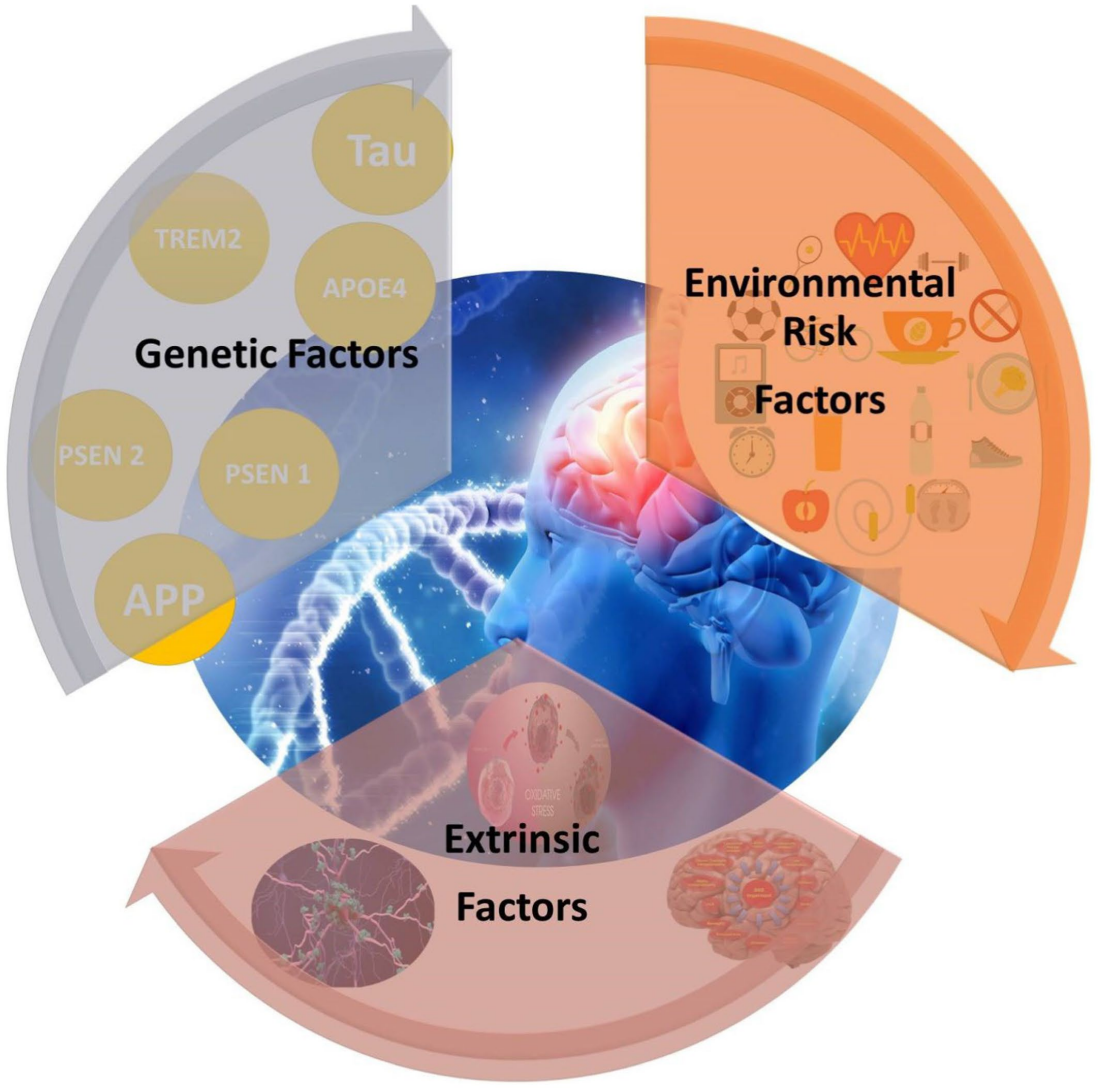
Pathological overview of Alzheimer’s disease.

Individual external factors such as food, cigarette smoking, exercise, and infections are included alongside macrolevel external factors such as rural versus urban life, exposure to pollution, and socioeconomic status in the proposed Alzheimer’s exposome. The restroom or toilet should be designed with the needs of people with dementia in mind. With the correct layout, a person with dementia can continue to take care of themselves with pride and autonomy. Store your mobile devices and other portable items somewhere secure to prevent them from going missing. Place smoke and carbon monoxide detectors in close proximity to the cooker and bedrooms. Battery life and operation should be regularly checked. Keep combustible and volatile materials away from gas appliances. The pathological overview of Alzheimer’s disease illustrated in Figure 4.

### 7.3 Vaccination and infectious agent management

Tdap/Td vaccine lowered the risk of AD by 30%, shingles vaccination reduced the risk by 25%, and pneumococcal vaccination reduced the risk by 27% when compared to those who did not receive the immunizations. The risk of developing AD appears to be reduced by vaccination against a wide variety of infectious agents (such as influenza, pneumococcus, and herpes zoster). This chapter examines the basic and pharmacoepidemiologic evidence for this association, paying special attention to important methodological variations among the epidemiologic studies, and reviews the remaining uncertainties regarding the effects of anti-pathogen vaccines on AD and all-cause dementia, including a discussion of the potential underlying mechanisms for this apparent protective effect of immunizations against infectious pathogens on the risk of AD (99).

### 7.4 Implications for public health policies

Memory loss and other cognitive impairments develop as AD progresses. Some of the issues that may arise include disorientation and wandering, difficulties with managing finances and paying bills, questions that need to be asked multiple times, delays in doing routine chores, and even changes in personality and behavior. The goal of public health is to promote everyone’s health and wellbeing over the lifespan. Most persons diagnosed with Alzheimer’s are over the age of 65, but the brain changes that precede the disease’s symptoms can begin years, even decades, earlier. A person’s likelihood of developing dementia in old age can be affected by their actions and medical history from much earlier in life. This is why public health interventions are required at all stages of life to combat AD and other forms of dementia. Common public health tools and techniques, such as early diagnosis and treatment plans, preventing harmful health practices, monitoring data collection, development of workforce competencies, and mobilization of partnerships across entire communities, can mitigate the future impact of AD.

## 8 Challenges and future directions

### 8.1 Limitations of current research

Due to the obstacles of the central nervous system, effective delivery and targeting of molecules of interest to the brain is another difficulty in treating AD. When the brain is the intended recipient, the blood–brain barrier (BBB) presents a challenge. Some of these symptoms include forgetfulness, slow language processing, trouble communicating, and shifts in mood. The principal impacts of the disease on a patient’s mind and body are accompanied by a significant shift in lifestyle and the ability to conduct routine chores. Inadequate training and resources for primary care physicians to conduct cognitive evaluations and send patients to research; cultural insensitivity and other impediments to inclusion for traditionally marginalized groups; the need for a study buddy.

### 8.2 The need for longitudinal studies and emerging research areas

The genetic analysis employed in the longitudinal study to assess the risk of developing Alzheimer’s disease focuses on the APOE e4 allele. One of the initial and significant discoveries in this study involves the difference in the rate of age-related memory decline between individuals carrying and those not carrying this allele. The gut bacteria’s direct production of neurotransmitters and neuromodulators, such as serotonin, dopamine, or short-chain fatty acids, has the potential to impact the functioning of the central nervous system. Intestinal enterochromaffin cells, responsible for producing various hormones and neurotransmitters, including serotonin, may be influenced by signals from the gut microbiota (100, 101). Disruptions in the brain-gut-microbiota axis may play a significant role in the development of Alzheimer’s disease (AD) and other neurodegenerative disorders. Dementia, marked by a gradual decline in cognitive abilities, is primarily caused by AD. The initial feasibility study of orally administered senolytic therapy in AD revealed promising safety data and dasatinib’s effective penetration into the brain. The drug demonstrated a moderate impact on Alzheimer’s and aging biomarkers (102, 103).

### 8.3 Potential therapeutic targets based on extrinsic factors

The amyloid cascade hypothesis suggested that beta-amyloid played a central role in the development of Alzheimer’s disease (AD). Numerous studies have extensively investigated the production, aggregation, and clearance of Aβ, focusing on both physiological and pathological processes. A potential breakthrough in AD treatment seems imminent with the identification of two crucial enzymes, γ-secretase and BACE1, responsible for cleaving the seemingly detrimental Aβ from its precursor (104). AChE, NMDAR, β-secretase, Aβ oligomers, Aβ plaques, GSK-3β, neurofibrillary tangles, Ca2+ ions, and reactive oxygen species are elements involved in the pathogenesis of Alzheimer’s disease (AD) and represent significant potential targets for treatment (105).

## 9 Conclusion

In conclusion, Alzheimer’s Disease (AD) is a result of the complex interaction between genetic predispositions and environmental influences. Genetic factors are crucial for early diagnosis, while lifestyle elements like diet, exercise, sleep, and exposure to contaminants significantly impact AD risk. Lifestyle changes, such as healthy eating, regular exercise, and quitting smoking, are fundamental preventive measures. Environmental risk reduction, optimizing living conditions, vaccination, and adapting surroundings for individuals with dementia are crucial. Public health policies should emphasize early diagnosis, health promotion, and community engagement to effectively combat AD’s impact on society. Implementing preventive health practices, monitoring, workforce development, and community collaboration can curb the looming impact of AD globally.

### 9.1 Implications for AD prevention and treatment

Families face challenges in providing care for AD patients due to conflicting needs and social problems. Early diagnosis and treatment can help maintain cognitive and functional abilities. Cognitive decline can be slowed with mental exercises and cholinesterase inhibitors. Healthy weight is achievable through balanced diet and exercise, benefiting brain health and emotional stability. Moderation in alcohol consumption can improve the quality of life for both patients and caregivers. Commonly used cholinesterase inhibitors include galantamine, rivastigmine, and donepezil, addressing mild to moderate Alzheimer’s symptoms.

### 9.2 Call to action for further research and initiatives

While environmental exposures are studied as potential AD risk factors, definitive evidence is lacking and requires more research. Genetic factors may influence susceptibility to environmental exposures, and lifestyle choices can interact with genetic and environmental factors, complicating disease development. Although links exist between AD risk and factors like air pollution and pesticides, conclusive evidence is lacking. Future research should prioritize understanding complex interactions and developing prevention strategies. Comprehending the interplay between genetic predisposition, lifestyle, and environmental exposures is crucial for effective AD prevention and treatment. Research should focus on unraveling mechanisms and identifying specific environmental factors while minimizing exposure to plastics, pollutants, and chemicals for overall health and well-being.

## Author contributions

SS: Conceptualization, Data curation, Formal analysis, Investigation, Methodology, Writing – review & editing. AS: Conceptualization, Data curation, Formal analysis, Investigation, Methodology, Writing – review & editing. RR: Conceptualization, Data curation, Formal analysis, Investigation, Methodology, Writing – review & editing. CV: Formal analysis, Supervision, Validation, Writing – review & editing. BP: Formal analysis, Supervision, Validation, Writing – review & editing.
